# Ethnomedicinal botany of the Apatani in the Eastern Himalayan region of India

**DOI:** 10.1186/1746-4269-1-11

**Published:** 2005-11-16

**Authors:** Chandra Prakash Kala

**Affiliations:** 1 GB Pant Institute of Himalayan Environment & Development, Kosi-Katarmal, Almora, Uttaranchal- 263 643, India

**Keywords:** Apatani tribe, Arunachal Pradesh, Eastern Himalaya, indigenous uses, ethnomedicinal plants

## Abstract

This paper investigates the wealth of medicinal plants used by the Apatani tribe of Arunachal Pradesh. Apatani have traditionally settled in seven villages in the Ziro valley of Lower Subansiri district of Arunachal Pradesh in the Eastern Himalayan region of India. The present study has resulted in the documentation of 158 medicinal plant species used by the Apatani group of villages. These medicinal plant species were distributed across 73 families and 124 genera. Asteraceae was the most dominant family (19 species, 11 genera) of medicinal plants, followed by Zingiberaceae, Solanaceae, Lamiaceae and Araceae. For curing ailments, the use of aboveground plant parts was higher (80%) than the belowground plant parts in the Apatani group of villages. Of the aboveground plant parts, leaf was used in the majority of cases (56 species), followed by fruit. Different belowground plant forms such as root, tuber, rhizome, bulb and pseudo-bulb were used by Apatani as a medicine. About 52 types of ailments were cured by using these 158 medicinal plant species. The results of this study are further discussed in the changing socio-economic contexts.

## Introduction

Tribal communities are mainly the forest dwellers who have accumulated a rich knowledge on the uses of various forests and forest products over the centuries. India possesses a total of 427 tribal communities, of these more than 130 major tribal communities live in North East India, which is comprised of the 8 states Meghalaya, Mizoram, Manipur, Tripura, Sikkim, Assam, Nagaland and Arunachal Pradesh. The major tribal communities of the North East India have been categorized into sub-tribes and if these sub-tribes are taken into account the total number of tribal groups reaches up to 300. In general, the tribes of North East India have been categorized into two broad ethnic communities, such as the Khasi and the Jaintia tribe of Meghalaya, who belong to 'Monkhemar' culture of Austoic dialect, and the rest of the tribal groups are basically Mongoloid, who belongs to Tibeto-Burman subfamily of Tibeto-Chinese group [1-3].

In the North East India, each state contains a number of tribal groups. Arunachal Pradesh is one of the states in North East India inhabited by 28 major tribes and 110 sub-tribes [4]. Arunachal Pradesh is the 12^th ^mega biodiversity region of the world [5]. More than 545 species of orchids have been reported from the state, which is the highest number of orchid species known from any single state of India [6]. Such a rich biodiversity in the state has provided an initial advantage to its inhabitants for observing, and scrutinizing the rich flora and fauna for developing their own traditional knowledge. Most of the tribe economies have been historically engaged in subsistence agriculture or hunting and gathering. Over the years, they have developed a great deal of knowledge on the use of plants and plant products in curing various ailments.

A review of the literature reveals that many tribal areas and tribal communities in the eastern Himalayan region of India are either under explored or unexplored with regard to their floral wealth used in curing diseases. The Apatani is one such little studied tribe of Arunachal Pradesh [3]. Therefore, a need was felt to gather in-depth information on the plant species used by this tribal group and suggest that similar studies need to be carried out across the various groups of tribes for comparison as well as for documenting the knowledge which may be under threat due to the influence of modernization. The present paper thus aims to highlight and record in detail the traditional knowledge of the Apatani tribe on the use of medicinal plant species growing in and around their settlements.

### Study area: Apatani group of villages

Literally, the word Apatani is composed of two words- 'Apa' and 'Tani'. According to the local language, 'Apa' means display of affection and 'Tani' stands for human race. The Apatani, generally, speak in their own language which has no script. Traditionally, they had settled in seven villages (e.g. Hong, Hari, Billa, Dutta, Hija, Mudang-Tage, and Michi Bamin) those were organized in accordance with the traditional lines of the three forefathers called Nichi-Nitii, Tinii-Diibo-Dre-Hija, and Talyang-Hao. These 7 villages are located in the Ziro valley of Lower Subansiri district in the central western part of Arunachal Pradesh in India between 26°55' – 28°21' N and 92°40' – 94°21' E. The Ziro valley (often called the Apatani valley) lies between the Panior and Kamla (Kuru) rivers at an altitude of 1524 to 2738 m a.m.s.l. The Apatani group of villages is located at 2200 m elevation. The Apatani migrated to the present location from the Talle Valley located in south eastern region [7]. The pattern of Apatani villages is that of string settlements (village houses are in a straight line) and homes are made of bamboo and timber. The Apatani belong to the Tibeto-Mongoloid stock, and trace their descent from one legendary ancestor, the Abotani.

The Apatani believe in indigenous religion called as 'Donyi-Polo' and are patriarchal in social system. The traditional village council, which regulates and administers the community, consists of three bodies namely *Akha Buliyang, Yapha Buliyang *and *Ajang Buliyang*. In each of these traditional institutions has one or two persons represent from each clan. Earlier, the Apatani had prominent tattoo marks on the face to distinguish themselves from other communities settled nearby. However, the practice of tattooing has been discouraged in the recent past and now is on the verge of extinction.

In 1991 census the population of Apatani was 22,526 (Table 1). The decadal (1991–2001) growth rate of the Apatani is 8.62%, which is much lower than that of the state (26.21%). The Ziro valley has an area of 1058 km^2 ^of which 43 km^2 ^is under agriculture, and remaining under forests, plantations and settlements. It is bounded with the areas traditionally belonging to neighbouring Nishi tribe. The land holding size varies from 0.02 to 10.00 ha with over 93% holdings consisting of 0.026–3.00 ha. The Ziro valley exhibits a humid sub-tropical to temperate type of climate with 108.1 cm rainfall and a temperature ranges from a maximum of 30.6°C to minimum of 1.1°C [8]. The climatic, altitudinal and geomorphological variations have shaped the two major vegetation types in and around the study area- sub-tropical forests and temperate forests. Sub-tropical forests in the study area are represented by *Castanopsis indica, Acer *sp.,* Pinus wallichiana *and *Pinus roxburghii*, whereas, the temperate forests are represented by *Quercus glauca, Alnus nepalensis, Castanopsis indica, Pyrus *sp.,* Prunus *sp.,* Populus *sp. and *Acer *sp [9,10]. Many shrub species such as *Berberis wallichiana, Viburnum foetidum, Prunus *sp., *Rubus *sp., *Spirea *sp. and *Symplocos *sp. occur in the forested areas.

**Table 1:** Demographic profile of the Apatani in Ziro valley of Arunachal Pradesh

**Year**	**Population**		**% to state**
		
	**Apatani**	**Arunachal Pradesh**	
1961	10,793	3,36,588	3.21
1971	12,888	4,68,511	2.75
1981	16,580	6,31,839	2.62
1991	22,526	8,64,558	2.61
2001	24,650	10,91,117	2.26

*Source*: Census of India, Part IX-B, Government of India.

### Medicinal plants survey

A literature survey was carried out for compilation of existing information on the medicinal plants used by Apatani villagers [2,3,10-14]. In addition, field surveys in Apatani villages were undertaken during May and June 2005 to gather data on the indigenous uses of medicinal plant species by the Apatani. During the survey period, information was also gathered using semi-structured questionnaires on types of ailments cured by the traditional use of medicinal plants and plant parts used in curing different ailments. Cross-checking of data was made with the help of group discussions among different age classes of Apatani villagers that include both the genders of the society. The participant observation method was also employed to understand the methods and techniques adopted by the Apatani in curing diseases. The surrounding forested area and agricultural land of the Apatani villages were also surveyed with local youths and knowledgeable elders for the identification of various medicinal plant species and their indigenous uses. Since there is lack of comprehensive records on floral diversity of North East Himalaya including Arunachal Pradesh, the plant specimens were identified through various floral inventories [10,13,15]. The collected information was analyzed, and correlation was made between different genera and species of the medicinal plants in order to understand the pattern in medicinal plant uses and occurrences.

## Results and discussion

The Apatani mainly subsist on agriculture and animal husbandry. Wet-rice cultivation is their most important agriculture practice. One of the Apatani proverbs reads "*Tanii hii jebi danii*", which means the Apatani depend on wet-rice cultivation. The Apatani have also developed a unique system of fish farming in their wet-rice croplands. They use available natural resources such as bamboo, cane, pine, *Phragmites *sp. and *Castanopsis *sp. in order to check the soil erosion, to conserve the soil fertility, to cultivate varieties of rice landraces, and to culture the fish in an integrated manner. Two species of bamboo (*Phyllostachys bambusoides *and *Dendrocalamus hamiltonii*) are also cultivated in private lands by the Apatani for construction of houses and other domestic uses. Bamboo shoots are also consumed by the Apatani as a vegetable. Apong, a locally prepared beer by fermenting rice, finger millet and barley, is an important beverage of the Apatani, which they prefer to consume with mutton. Domestic and semi-domestic cattle also play important role in maintaining the economic status of the Apatani. Possessing a large number of domestic animals is an indication of the prosperity of their respective owner [16]. Mithun (*Bos frontalis*) is preferred mostly for the meat. In addition, pigs, cows, and multiple varieties of birds and fish are consumed by the Apatani. A number of wild edible fruits and vegetables are also collected by the Apatani from the nearby forested areas to supplement the domestic nutritional requirements.

Traditionally, the Apatani group of villages was not only familiar with the knowledge of medicinal plants but they were also expert traders and met their necessities in exchange of paddy, which was always in excess of their requirements [17]. Earlier, they had no connection with the plains of Assam due to obstructions created by the Nishi who were earning a lot by acting as middlemen between the Apatani and the people residing in the plains. However, the Apatani had occupied a compact area in Ziro valley and were one of the self-sufficient tribes in North East India [8]. Their immediate dependence on nature had developed knowledge which ultimately is reflected in their traditional culture, religion, local belief, folklore, taboos language and dialects. For many centuries, the Apatani had kept alive a self-managed system of folk medicine that was mainly based on herbal remedies [10]. Their ingenuity still reflects their traditional management and sharing of natural resources in a way that there is optimum utilization of such resources [8,18]. The Nishi are one of the neighbours of the Apatani who live at lower elevations and are the most populous tribe in the state. Over the past few decades, the interaction between the Apatani and the Nishi has increased many fold due to migration of Apatani people in search of better education in Itanagar, a capital of Arunachal Pradesh. The availability of motor roads and the invasion of modern civilization have also enhanced the day to day interaction and the exposure of the Apatani to the rest of the world. Such interaction has provided a possible sharing of traditional knowledge of the Apatani with their neighbouring community.

**Figure 1:**
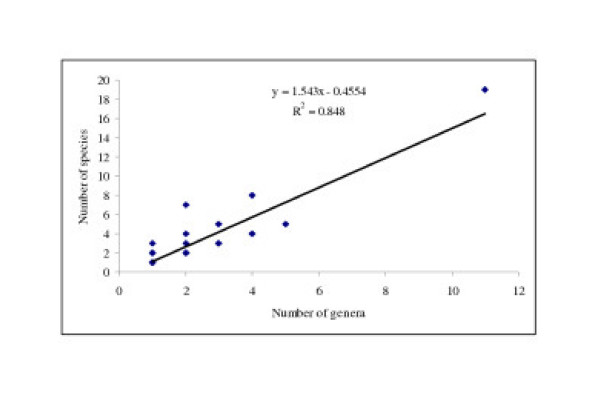
Relationship between genera and species richness of medicinal plants used by the Apatani in Ziro valley of Arunachal Pradesh.

During the present course of investigations, a total of 158 medicinal plant species used by the Apatani group of villages were documented. These medicinal plant species were distributed across 73 families and 124 genera (Table 2). In terms of number of medicinal plant species, Asteraceae was the most dominant family (19 species, 11 genera) of medicinal plants, followed by Zingiberaceae, Solanaceae, Lamiaceae, Araceae, and Verbanaceae (Table 3). There was a significant positive correlation (r = 0.92, p > 0.01) between the number of genera and number of species used as medicine by the Apatani (Figure 1). The invention of maximum number of uses of Asteraceae by the Apatani tribe demonstrates the dominance of Asteraceae around the Apatani group of villages. Asteraceae is the most dominant family of medicinal plants across the North Eastern States of India [13].

**Table 2:** Medicinal plant species, plant parts used and ailments cured by the Apatani of Ziro valley in Arunachal Pradesh

**Sl No.**	**Species**	**Family**	**Part used**	**Uses**
1	*Acorus calamus *L.	Araceae	Root	Cut, wounds, skin diseases, bone fracture
2	*Ageratum conyzoides *L.	Asteraceae	Leaf	Cut, wounds
3	*Allium cepa *L.	Liliaceae	Bulb	Eye pain
4	*Allium hookeri *Thwait.	Liliaceae	Bulb	Eruption of skin, cough, cold, wounds
5	*Alocasia forniculata *(Roxb.) Schott.	Araceae	Root	Crack of heels
6	*Alstonia scholaris *(L.) Br.	Apocynaceae	Leaf, bark	Headache, stomach disorder, menstrual disorder
7	*Amomum aromaticum *Roxb.	Zingiberaceae	Leaf, seed	Fever, abortion
8	*Amorphophallus paeoniifolius *(Dennst.) Nicolson	Araceae	Corn	Piles
9	*Andrographis paniculata *(Burm. f.) Wall. ex Nees	Acanthaceae	Leaf	Dysentery
10	*Anisomeles indica *(L.) O.K.	Lamiaceae	Shoot	Bodyache
11	*Angiopteris evecta *(Forst.) Hoffm.	Angiopteridaceae	Stem	Health tonic
12	*Antidesma acidum *Retz.	Euphorbiaceae	Leaf	Wounds
13	*Argemone mexicana *L.	Papaveraceae	Shoot	Skin diseases
14	*Artemisia indica *Willd.	Asteraceae	Leaf	Bodyache, asthma, skin diseases
15	*Artemisia maritima *L.	Asteraceae	Shoot	Blood purification
16	*Artemisia nilagirica *(Cl.) Pamp.	Asteraceae	Leaf	Cough, headache, sores
17	*Asplenium nidus *L.	Aspleniaceae	Leaf	Ulcer
18	*Barleria prionitis *L.	Acanthaceae	Leaf	Cough
19	*Begonia roxburghii *(Miq.) DC.	Begoniaceae	Leaf	Indigestion
20	*Berberis wallichiana *(Wall.) Brongn.	Berberidaceae	Fruit, root	Indigestion, bodyache
21	*Bergenia ciliata *(Haw.) Sternb.	Saxifragaceae	Root, leaf	Cut, wounds
22	*Brassiopsis glomarulata *(Bl.) Regel.	Araliaceae	Fruit	Cough
23	*Buddleja asiatica *Lour.	Buddlejaceae	Leaf	Inflammation
24	*Callicarpa macrophylla *Vahl	Verbenaceae	Leaf	Headache
25	*Callicarpa vastita *Roxb.	Verbenaceae	Leaf	Indigestion
26	*Calotropis gigantea *(L.) Br.	Asclepiadaceae	Root	Dog bite
27	*Canarium resiniferum *Brace ex King	Burseraceae	Fruit	Urinary complaints
28	*Capparis spinosa *Lam.	Capparaceae	Root	Rheumatic pain
29	*Cardamine hirsuta *L.	Brassicaceae	Leaf	Indigestion
30	*Castanopsis tribuloides *(Sm.) DC.	Fagaceae	Stem	Cough, goiter, indigestion
31	*Centella asiatica *L.	Apiaceae	Shoot	Constipation, gastritis, blood purification
32	*Chenopodium ambrosioides *L.	Chenopodiaceae	Leaf	Toothache
33	*Christella parasitica *(L.) Lev.	Thelypteridaceae	Fronds	Cut, wounds
34	*Chromolaena odorata *(L.) King & Robinson	Asteraceae	Leaf	Cut, wounds, headache, fever
35	*Cirsium lapskyle *Petral.	Asteraceae	Shoot	Indigestion
36	*Cissampelos pareira *L.	Menispermaceae	Tuber	Health tonic
37	*Clerodendrum glandulosum *Coleb. ex Wall.	Verbenaceae	Leaf	Blood pressure, fever, cough
38	*Clerodendrum serratum *(L.) Moonb	Verbenaceae	Leaf	Eye disorders
39	*Coelogyne pectata *Lindl.	Orchidaceae	Pseudobulb	Burns
40	*Colocasia affinis *Schott	Araceae	Leaf	Fever, respiratory disorder
41	*Crassocephalum crepidioides *(Benth.) Moore	Asteraceae	Leaf	Indigestion, headache, stomachache, cut, wounds
42	*Crotolaria pallida *Ait.	Fabaceae	Root	Bodyache
43	*Croton roxburghii *Balak	Euphorbiaceae	Fruit	Indigestion
44	*Curcuma caesia *Roxb.	Zingiberaceae	Rhizome	Cough, asthma
45	*Curcuma aromatica *Salisb.	Zingiberaceae	Whole plant	Blood purification
46	*Curcuma zedoaria *Rosc.	Zingiberaceae	Rhizome	Cold, cough
47	*Cuscuta reflexa *Roxb.	Cuscutaceae	Whole plant	Purgative
48	*Cyathea gigantea *(Wall. ex Hk. f.) Holt.	Cyatheaceae	Leaf	Bodyache
49	*Cyathula prostrata *(L.) Bl.	Amaranthaceae	Shoot	Appetizer, dysentery, skin diseases
50	*Cymbidium aloifolium *(L.) Sw.	Orchidaceae	Tuber	Wounds
51	*Dendrocnide sinuta *(Bl.) Chew.	Urticaceae	Leaf	Urogenital disorder, toothache, dysentery
52	*Dicranopteris linearis *(Burm. f.) Und.	Gleicheniaceae	Whole plant	Indigestion
53	*Dicrocephala bicolor *(Roth) Sch.	Asteraceae	Shoot	Digestive problems
54	*Dillenia indica *L.	Dilleniaceae	Furit	Stomachache
55	*Dioscorea alata *L.	Dioscoraceae	Tuber	Indigestion
56	*Dioscorea bulbifera *L.	Dioscoraceae	Tuber	Indigestion
57	*Dioscorea hamiltonii *Hk. f.	Dioscoraceae	Tuber	Dysentery
58	*Diplazium esculentum *(Retz.) Sw.	Athyriaceae	Fronds	Constipation
59	*Ecbolium viride *(Forsk) Alston	Meliaceae	Root	Rheumatism
60	*Eclipta prostrata *(L.) L.	Asteraceae	Shoot	Cut, wounds
61	*Elaeagnus caudata *Sch. ex Momiyama	Elaeagnaceae	Fruit	Health tonic
62	*Elaeagnus pyriformis *Hk. f.	Elaeagnaceae	Fruit	Constipation
63	*Elatostema platyphyllum *Wedd.	Urticaceae	Root	Vomiting
64	*Elsholzia blanda *(Benth.) Benth.	Lamiaceae	Leaf	Itching
65	*Eluesine coracana *(L.) Gaertn.	Poaceae	Grains	Stomach disorder, tonic, cold
66	*Eupatorium odoratum *L.	Asteraceae	Leaf	Wounds, cut
67	*Erigeron bonariensis *L.	Asteraceae	Leaf	Nose block
68	*Eryngium foetidum *L.	Apiaceae	Seed	Madness, headache
69	*Ficus benjamina *L.	Moraceae	Stem	Stomach disorder
70	*Ficus hirta *Vahl	Moraceae	Fruit	Wounds, cut
71	*Gerbera pilosellioides *(L.) Cass.	Asteraceae	Leaf	Rheumatic pain
72	*Gloriosa superba *L.	Liliaceae	Tuber	Killing lice in hairs
73	*Gmelina arborea *Roxb.	Verbenaceae	Leaf	Stomach disorder
74	*Gynostemma pedata *Bl.	Cucurbitaceae	Leaf	Throat ache
75	*Gynura biscolor *(Roxb. ex Willd.) DC.	Asteraceae	Leaf	Intestinal worms
76	*Gynura nepalensis *DC.	Asteraceae	Leaf	Indigestion
77	*Hedychium coronarium *Koen.	Zingiberaceae	Rhizome	Bodyache
78	*Hedychium dekianum *Rao & Verma	Zingiberaceae	Rhizome	Cut, wounds
79	*Hedychium spicatum *Buch.-Ham. ex Sm.	Zingiberaceae	Rhizome	Stomach disorder
80	*Hibiscus rosa-sinensis *L.	Malvaceae	Flower	Reproductive disorders
81	*Houttuynia cordata *Thunb.	Saururaceae	Shoot	Freshness, good sleep, heart disorders
82	*Hyptis suaveolens *(L.) Poit.	Lamiaceae	Leaf	Itching, cough, cold
83	*Hypericum japonicum *Thunb. ex Murr.	Hypericaceae	Stem	Cut, wounds
84	*Impatiens latifolia *L.	Balsaminaceae	Leaf	Headache, digestive disorder
85	*Impatiens racemosa *DC.	Balsaminaceae	Leaf	Digestive disorder
86	*Indigofera tinctoria *L.	Fabaceae	Root	Wound
87	*Jasminum humile *L.	Oleaceae	Root	Ringworm
88	*Laginaria siceraria *(Molina) Standl.	Cucurbitaceae	Fruit	Burns
89	*Leonotis nepetifolia *R. Br.	Lamiaceae	Seed	Burns
90	*Lithocarpus dealbatus *(Miq.) Rehder	Fagaceae	Fruit	Indigestion
91	*Litsea cubeba *(Lour.) Pers.	Lauraceae	Fruit	Cough, cold, hair tonic, indigestion, good sleep
92	*Litsea salicifolia *(Nees) Hk.f.	Lauraceae	Fruit	Bone fracture, stomach disorder
93	*Lygodium scandens *(L.) Sw.	Schizaeaceae	Leaf	Skin diseases
94	*Mahonia napalensis *DC.	Berberidaceae	Stem	Itching
95	*Measa indica *(Roxb.) DC.	Myrsinaceae	Fruit	Indigestion
96	*Mikania micrantha *Kunth.	Asteraceae	Leaf	Itching, skin diseases, headache
97	*Miliusa roxburghiana *(Wall. ex Griff.) Hk. f. & Th.	Annonaceae	Leaf	Headache
98	*Molineria crassifolia *Baker	Hypoxidaceae	Fruit	Diarrhoea
99	*Molineria recurveta *(Dryand) Hebbert.	Hypoxidaceae	Leaf	Bodyache
100	*Mucuna pruriens *(L.) DC.	Fabaceae	Stem	Eye disorder
101	*Murraya koenigii *(L.) Spr.	Rutaceae	Leaf	Stomach trouble
102	*Musa paradissica *L.	Musaceae	Fruit	Indigestion
103	*Myrica esculenta *Ham. ex D. Don.	Myricaceae	Fruit, bark	Indigestion, skin eruption
104	*Myrsine semiserrata *Wall.	Myrsinaceae	Seed	Skin diseases
105	*Oenanthe javanica *(Bl.) DC.	Apiaceae	Shoot	Indigestion
106	*Oroxylum indicum *(L.) Vent.	Bignoniaceae	Seed	Purgative, headache
107	*Osbeckia stellata *Buch.-Ham. ex D. Don	Melastomataceae	Leaf	Toothache
108	*Oxalis corniculata *L.	Oxalidaceae	Shoot	Appetizer, headache
109	*Paedaria foetida *L	Rubiaceae	Stem	Gastritis, diarrhea, stomach disorder
110	*Passiflora foetida *L.	Passifloraceae	Fruit	Respiratory disorder
111	*Photinia integrifolia *Lindl.	Rosaceae	Fruit	Indigestion
112	*Perilla frutescens *(L.) Britt.	Lamiaceae	Seed	Fever, headache
113	*Physalis angulata *L.	Solanaceae	Fruit	Gastric trouble
114	*Physalis minima *L.	Solanaceae	Fruit	Gastric trouble
115	*Physalis peruviana *L.	Solanaceae	Leaf	Pain in pregnancy
116	*Picrorhiza kurrooa *Benth.	Scrophulariaceae	Root	Cold, fever
117	*Pinus roxburghii *Sarg.	Pinaceae	Seed	Indigestion
118	*Piper brachystachyum *Wall.	Piperaceae	Seed	Cough
119	*Piper trioicum *Roxb.	Piperaceae	Root	Cough
120	*Plantago major *L.	Plantaginaceae	Leaf	Constipation
121	*Plectranthus japonicus *(Burm. f.) Koidz.	Acanthaceae	Leaf	Fever
122	*Polygonum nepalense *Meissn.	Polygonaceae	Leaf	Indigestion
123	*Polygonum perfoliatum *L.	Polygonaceae	Leaf	Indigestion
124	*Portulaca oleracea *L.	Portulacaceae	Stem, leaf	Appetizer
125	*Pouzolzia hirta *(Bl.) Hassk.	Urticaceae	Root	Constipation
126	*Pterospermum acerifolium *Willd.	Sterculiaceae	Flower	Earache
127	*Rhus chinensis *Miller	Anacardiaceae	Fruit	Blood dysentery
128	*Rubia cordifolia *L.	Rubiaceae	Shoot	Stomachache
129	*Rubus calycinus *Wall.	Rosaceae	Fruit	Stomach disorder
130	*Rubus ellipticus *Sm.	Rosaceae	Fruit	Indigestion
131	*Rubus paniculatus *Sm.	Rosaceae	Fruit	Stomach disorder
132	*Rubus roseafolius *Sm.	Rosaceae	Fruit	Indigestion
133	*Rumex nepalensis *Spr.	Polygonaceae	Leaf	Indigestion
134	*Saurauria roxburghii *Wall.	Saurauriaceae	Leaf	Constipation
135	*Schefflera glomerata *L.	Araliaceae	Fruit	Indigestion
136	*Schizostachium capitatum *(Munro) Majumdar	Poaceae	Shoot	Diarrhea, dysentery, stomach disorder
137	*Senna alata *(L.) Roxb.	Caesalpiniaceae	Leaf	Skin diseases
138	*Senna tora *(L.) Roxb.	Ceasalpiniaceae	Leaf	Low blood pressure
139	*Sphenomeris chinensis *(L.) Maxon	Lindsaeceae	Fronds	Sprains
140	*Solanum kurzii *Brace ex Prain	Solanaceae	Fruit	Cough, worms infestation
141	*Solanum myriacanthum *Dunal	Solanaceae	Seeds	Toothache
142	*Solanum nigrum *L.	Solanaceae	Leaf	Liver tonic, indigestion
143	*Solanum torvum *Sm.	Solanaceae	Fruit	Cough, skin diseases
144	*Sonchus asper *(L.) Hill	Asteraceae	Shoot	Indigestion
145	*Sonchus arvensis *L.	Asteraceae	Shoot	Stomachache, gastritis
146	*Spilanthus clava *L.	Asteraceae	Leaf	Throat pain
147	*Spilanthes paniculata *DC.	Asteraceae	Leaf	Constipation
148	*Stellaria media *(L.) Vill.	Caryophyllaceae	Leaf	Itching
149	*Stereospermum chelonoides *(L. f.) DC.	Bignoniaceae	Leaf	Sprain
150	*Strobilanthus helictus *T. Anders.	Acanthaceae	Shoot	Indigestion
151	*Terminalia chebula *Retz.	Combretaceae	Fruit	Cough
152	*Toddalia aculeata *Pers.	Rutaceae	Fruit	Throat pain
153	*Urtica dioica *L.	Urticaceae	Leaf	Bone fracture
154	*Vernonia cinerea *(L.) Less	Asteraceae	Leaf	Indigestion
155	*Zanthoxylum acanthopodium *DC.	Rutaceae	Fruit	Dysentery
156	*Zanthoxylum armatum *DC.	Rutaceae	Fruit	Cold, cough, fever, appetizer
157	*Zanthoxylum oxyphyllum *Edgew.	Rutaceae	Fruit	Stomach disorder
158	*Zingiber officinale *Rosc.	Zingiberaceae	Rhizome	Cough

**Table 3:** Dominant families of medicinal plants used by the Apatani in terms of number of species occupied

**Family**	**Genera**	**Species**
Asteraceae	11	19
Zingiberaceae	4	8
Solanaceae	2	7
Lamiaceae	5	5
Araceae	5	5
Verbenaceae	3	5
Rutaceae	3	5
Rosaceae	2	5
Urticaceae	4	4
Acanthaceae	4	4

Different parts of medicinal plant species were used by the Apatani as a medicine. For curing ailments, the use of aboveground plant parts was higher (80%) than the belowground plant parts. Of the aboveground plant parts, leaf was used in the majority of cases (56 species), followed by fruits. Different belowground plant forms such as root, tuber, rhizome, bulb and pseudo-bulb were also used by the Apatani as a source of curing ailments (Table 4). The whole plant of 3 species [e.g. *Curcuma aromatica *Salisb., *Cuscuta reflexa *Roxb. and *Dicranopteris linearis *(Burm. f.) Und.] was used as medicine. These 158 medicinal plant species were used in curing about 52 types of ailments, of which the highest numbers of plant species (40 species) were used for the treatment of gastrointestinal disorders such as indigestion and constipation. About 19 medicinal plant species were used in curing cough and cold, and 15 medicinal plant species were used for healing cuts and wounds (Table 5).

**Table 4:** Patterns in Apatani use of medicinal plant parts

**Aboveground plant parts used**	**Number of Species**	**Belowground plant parts used**	**Number of Species**
Whole shoot	15	Root	14
Leaf	56	Tuber	6
Fruit	31	Rhizome	6
Seed	10	Bulb	2
Stem	6	Pseudo-bulb	1
Fronds	4	Corn	1
Bark	2		
Flower	2		
**Total**	**126**	**Total**	**30**

**Table 5:** Major ailments cured by the Apatani in terms of using the plant species

**Ailments**	**Number of plants used**
Indigestion	40
Cough and cold	19
Cut and wounds	15
Headache	12
Stomach disorder	11
Skin diseases	11
Fever	8
Body-ache	6
Dysentery	6
Throat-ache	5

Previous studies carried out in North East India have reported 41 medicinal plant species used by the Apatani of Arunachal Pradesh [13]. However, they had selected many North Eastern States and 12 different tribal communities for investigations. Based on their experiences, they had suggested the need of carrying out detailed investigations of each tribe. So far different authors have reported 1350 species of plants used in ethnomedicinal preparations, 665 species of food plants and 899 species for miscellaneous uses from the entire North East India [3]. The present inventory of 158 medicinal plant species as used by the Apatani is one of its kinds in terms of the highest number of species recorded so far used by a single tribe of the North East India. This fact provides a strength to the statements of earlier researchers that North East India is still under-explored and certain areas in the district of Subansiri still remain unexplored [3,10]. Hence, a need for detailed investigations of ethnobotanical knowledge held by each tribal community in North East India is required before such valuable knowledge vanishes. In spite of the rich wealth of bio-resources and potential, development is far from meeting the expectations of local people in Arunachal Pradesh mainly in terms of existing health care facilities and herbal industries. Ethnomedicinal knowledge is also important from a humanitarian point of view in that in long run as this knowledge may help to identify important medicinal uses that can help in curing and healthcare around the world. Attempts should be made to share the benefits arising from such knowledge with its holders. The present inventory of medicinal plants used by the Apatani opens new avenues to scrutinize such a rich natural resource for further analysis in order to develop the potential of herbal medicine.
